# Development of Novel Magneto-Biosensor for Sulfapyridine Detection

**DOI:** 10.3390/bios10040043

**Published:** 2020-04-21

**Authors:** Talha Jamshaid, Ernandes Taveira Tenório-Neto, Abdoullatif Baraket, Noureddine Lebaz, Abdelhamid Elaissari, Ana Sanchís, J.-Pablo Salvador, M.-Pilar Marco, Joan Bausells, Abdelhamid Errachid, Nadia Zine

**Affiliations:** 1 Institut des Sciences Analytiques, University Claude Bernard Lyon-1, UMR 5280, 5 rue de la Doua, F-69100 Villeurbanne, France; talhajamshaid007@gmail.com (T.J.); a.baraket@gmail.com (A.B.); Nadia.zine@univ-lyon1.fr (N.Z.); 2 University Claude Bernard Lyon-1, CNRS, LAGEPP UMR-5007, 43 Boulevard du 11 Novembre 1918, F-69100 Villeurbanne, France; tenorioernandes@gmail.com (E.T.T.-N.); noureddine.lebaz@univ-lyon1.fr (N.L.); 3 Faculty of Pharmacy and Alternatives Medicins, The Islamia University of Bahawalpur, Bahawalpur 63100, Pakistan; 4 Department of Chemistry, State University of Ponta Grossa, Av. Gen. Carlos Cavalcanti, 4748, CEP 84030-900 Ponta Grossa, Paraná, Brazil; 5 Nanobiotechnology for Diagnostics (Nb4D), Department of Chemical and Biomolecular Nanotechnology, Institute for Advanced Chemistry of Catalonia (IQAC) of the Spanish Council for Scientific Research (CSIC), Jordi Girona 18-26, 08034 Barcelona, Spain; asvnqb@iqac.csic.es (A.S.); jpablo.salvador@iqac.csic.es (J.-P.S.); pilar.marco@cid.csic.es (M.-P.M.); 6 CIBER de Bioingeniería, Biomateriales y Nanomedicina (CIBER-BBN), Jordi Girona 18-26, 08034 Barcelona, Spain; 7 Institute of Microelectronics of Barcelona (IMB−CSIC), Universitat Autònoma de Barcelona (UAB), Bellaterra, 08193 Barcelona, Spain; joan.bausells@imb-cnm.csic.es

**Keywords:** biosensor, sulfapyridine, SA2BSA, BioMEMS, magnetic nanoparticles, competitive assay

## Abstract

In this work, we report the development of a highly sensitive biosensor for sulfapyridine detection based on an integrated bio micro-electromechanical system (Bio-MEMS) containing four gold working electrodes (WEs), a platinum counter electrode (CE), and a reference electrode (RE). Firstly, the cleaned WEs were modified with 4-aminophenylacetic acid (CMA). Then, (5-[4-(amino)phenylsulfonamide]-5-oxopentanoic acid (SA2BSA) was immobilized onto the transducers surface by carbodiimide chemistry. The analyte was quantified by competitive detection with SA2BSA immobilized on the WE toward a mixture of Ab155 antibody (with fixed concentration) and sulfapyridine. In order to obtain a highly sensitive biosensor, Ab155 was immobilized onto magnetic latex nanoparticles surface to create a 3D architecture (Ab-MLNp). Using electrochemical impedance spectroscopy (EIS), we investigated the influence of the Ab-MLNp on the sensitivity of our approach. The optimized system was analyzed, as competitive assay, with different concentrations of sulfapyridine (40 µM, 4 µM, and 2 nM) and with phosphate buffer solution. From data fitting calculations and graphs, it was observed that the EIS showed more linearity when Ab-MLNp was used. This result indicates that the magnetic latex nanoparticles increased the sensitivity of the biosensor.

## 1. Introduction

Increasing attention has been paid to antibiotics as aquatic micropollutants with their environmental fate and impact to be understood [1]. Sulfonamide antibiotics (SAs), as one of the most important classes of antibiotics, are widely used in aquaculture, livestock husbandry, and human medicine. Recently, SAs were detected ubiquitously in the aquatic environment, which may pose risks toward organisms [2,3,4]. Among the SAs, sulfapyridine, which is commonly used in aquaculture, was frequently detected in various environmental waters (e.g., wastewater effluents and receiving water bodies as well as fish farms and adjacent water bodies) [5]. For the detection of sulfapyridine, various methods have been used, such as chromatographic methods (likely high-performance liquid chromatography coupled with mass spectrometric detection (HPLC-MS)). Such methods have been applied due to their sensitivity and compound quantification data. Sample preparation is required using commercially available cartridges for solid-phase extraction. Additionally, other techniques have been employed, such as thin layer chromatography, gas chromatography (GC), liquid chromatography (LC) (including their variations coupled with mass spectrometry), and radio-active immune receptor for purpose of foodstuffs [6,7,8]. However, the above-mentioned approaches are time consuming and require complex sample preparation procedures, expensive laboratory equipment, and skilled professionals to handle these techniques. 

In this sense, biosensors may offer cost-effective solutions for analyte detection. The biosensor is a compact analytical device or unit incorporating a biological (or biologically) derived sensitive element associated with a physicochemical transducer. They have revolutionized modern analysis due to their technical simplicity, low cost, and the possibility of being employed in field analysis [9,10]. The detection of sulfonamides using biosensors was previously demonstrated in different works [11,12,13]. However, few examples can be found using impedance spectroscopy for SA detection.

To improve the biosensor sensitivity, recently, magnetic nanoparticles (MNP) were produced as labels for biosensing. For the biosensing purpose, different types of biosensors were produced, such as giant-magnetoresistive (GMR) sensors and spin valves (SV) cantilevers [14,15], inductive sensors [16], superconducting quantum interference devices (SQUIDs) [17], anisotropic-magnetoresistive (AMR) rings, and miniature Hall crosses [18]. The detection of biological molecules is usually achieved by using biomolecular recognition between the target molecule and a specific receptor as, for example, an antibody that is tagged with a label.

In this context, superparamagnetic iron oxide nanoparticles (SPIONs) have been used as carriers for immobilization of biomolecules, such as peptides, proteins, and antibodies, to enhance the specific capture of the targeted biomolecules [19]. For preparing structured SPIONs with well-defined surface properties, specific functional groups, and better colloidal stability, several approaches have been investigated, including seeded-emulsion polymerization [20,21]. Using such an approach, magnetic latex nanoparticles with high iron oxide content can be obtained. These particles have reactive functional groups to form conjugates with various biomolecules (e.g., proteins, antibodies, DNA, and so forth), making them promising candidates to improve automation. Furthermore, the magnetic latex particles can enhance the diagnosis sensitivity by increasing the concentration of the captured targets [22].

Herein, an alternative procedure for sulfapyridine detection is proposed. Our approach consists of the development of bio-micro-electro-mechanical system (Bio-MEMS) transducers based on four gold micro-working electrodes (µWE) with fully integrated reference (RE) and platinum counter electrodes (CE). The surface of µWE was modified with coating antigen (5-[4-(amino)phenylsulfonamide]-5-oxopentanoic acid (SA2BSA), and the quantification of sulfapyridine was achieved through competitive assay toward antibodies Ab-155 deposited onto the magnetic latex nanoparticles surface. In addition, to evidence our proof of concept, we also compared the results obtained from our approach with those obtained from the competitive detection without magnetic latex particles.

## 2. Materials and Methods

### 2.1. Reagents and Apparatus

The 4-aminophenylacetic acid 98%, the N-Hydroxysuccinimide (NHS), and the 1-Ethyl-3-(3-dimethylaminopropyl)carbodiimide (EDC) were purchased from Acros Organics. Sodium nitrite was acquired from Fisher Scientific. Triton X-405 and phosphate buffer saline tablet were acquired from Sigma, ethanolamine from Fluka analytical, and hydrochloric acid 37% (HCl) from VWR, France. Iron (II) chloride tetrahydrate (FeCl_2_·4H_2_O), iron (III) chloride hexahydrate (FeCl_3_·6H_2_O), and oleic acid were purchased from Merck. Octane, ammonium hydroxide, and chlorhydric acid were from Prolabo. The preparation of the described bioconjugates and antibodies (5-[4-(amino)phenylsulfonamide]-5-oxopentanoic acid (SA2BSA) and Ab155) was performed with the support of the ICTS (Infraestructuras Científico-Tecnológicas Singulares “NANBIOSIS” Spain), more specifically by the Custom Antibody Service (CAbS, CIBER-BBN, IQAC-CSIC). The immunoreagents for sulfapyridine (SPy) detection used for the development of the biosensor were described before [23]. All other chemicals were purchased from Sigma-Aldrich and were used as received. All electrochemical measurements were performed on a VMP3 electrochemical workstation.

### 2.2. Preparation of Oil-in-Water Magnetic Emulsion

The oil-in-water magnetic emulsion (ME) consisted of magnetite nanoparticles stabilized with oleic acid, octane, and dodecyl sodium sulfate and was prepared according to our previous work [20,24,25]. Before obtaining the ME, the superparamagnetic iron oxide nanoparticles (SPIONs) were synthetized by coprecipitation method. Firstly, a solution containing iron chloride salts (ratio [Fe^3+^]/[Fe^2+^] = 2) was prepared. Then, concentrated ammonia was carefully added to this solution to produce magnetic nanoparticles. When the solution pH was around 9, oleic acid (stabilizing agent) was added to the dispersion under vigorous stirring. The SPIONs were extracted by introducing octane (organic phase) in the medium. Finally, the organic ferrofluid was collected by magnetic separation and filtered on a 1 mm porous nylon filter in order to eliminate aggregates, and it was concentrated by octane evaporation under vacuum until it achieved a solid content of about 70% wt.

Afterward, the organic ferrofluid was emulsified in an aqueous solution of a nonionic surfactant (Triton X-405, at least 25 wt% in water) and sheared in a couette emulsifier in order to target submicron ferrofluid droplets. The sheared emulsion was recovered and then diluted in a water solution containing sodium dodecyl sulphate (SDS) (2 g L^−1^). Then, the oil-in-water magnetic emulsion was sorted under magnetic field in order to collect narrowly sized distributed droplets. Lastly, the magnetic emulsion was diluted until it achieved a total solid content of about 4% wt.

### 2.3. Electrochemical Measurements

The electrochemical measurements were made by using a multi-channel potentiostat (VMP3 Biologic Science Instrumentation, France). Cyclic voltammetry (CV) and electrochemical impedance spectroscopy (EIS) were applied to characterize the surface of WEs before and after bio-functionalization using K_3_[Fe(CN)_6_]/K_4_[Fe(CN)_6_] (5 mM) as the electrolyte in phosphate buffered saline (PBS, pH 7.4). CV measurements were made at potentials of 0.5 V to −0.3 V, scan rate of 80 mV s^−1^, and 3 cycles. EIS measurements were made at a potential of 0.228 V, frequency of 200 kHz to 200 mHz, and sinus amplitude of 25 mV.

### 2.4. Fabrication of the Biosensor

The Bio-MEMS, based on gold microelectrodes, was fabricated in collaboration with the National Centre of Microelectronic (CNM) in Spain. The detailed process of fabrication and the characterization of the microlectrode were previously published in our group [26]. Briefly, a silicon wafer of 100 mm diameter was used as a substrate. Silicon dioxide (SiO_2_) was then grown up to 800 nm by wet thermal oxidation. Then, gold working microelectrodes were defined by deposition and patterning of a metal tri-layer of Ti (50 nm), Ni (50 nm), and Au (200 nm). For the counter microelectrode, a bilayer of Ti (15 nm) plus Pt (150 nm) was deposited. Finally, for the reference integrated microelectrode, a bilayer of Ti (15 nm) plus Ag (150 nm) was deposited and patterned by lift-off. All microelectrodes dimensions and the process of fabrication were detailed in our previous work [26].

**Figure 1:**
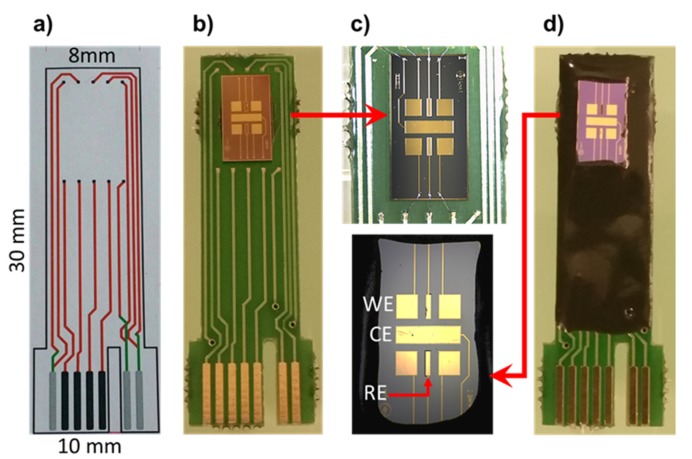
Optical microscope images of (**a**) printed circuit board (PCB) dimensions, (**b**) transducers glued onto the PCB board, (**c**) electrical connections, and (**d**) passivation with epoxy resin

After the fabrication process, the wafer was diced to individual devices 4 × 7 mm and was then glued to the PCB board (Figure 1b). Micro pads of all microelectrodes were then electrically connected to the PCB board (Figure 1c) and then were passivated with epoxy resin (Ep-Tek H70E-2LC) to protect all electrical connections (Figure 1c) [26].

Working electrodes (WEs) were cleaned by rinsing them with ethanol for 5 minutes, and then they were properly rinsed with deionized water. They were then dried under steam of nitrogen. The surface WEs were then cleaned from organic contaminants by using UV/O_3_ (UV/Ozone ProCleanerTM, BioForce Nanosciences) for 30 min. The device was connected to the potentiostat VMP3. Analysis was monitored and modeled using the EC-Lab software.

**Figure 2:**
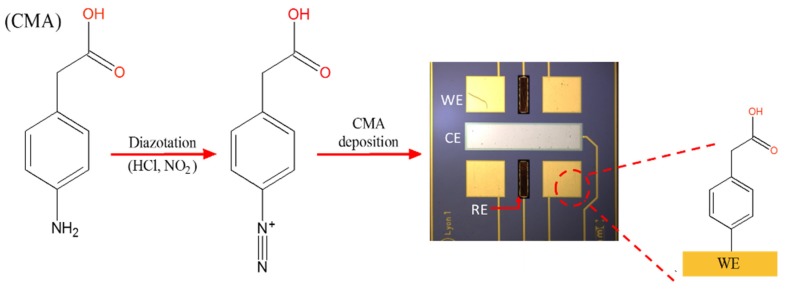
Schematic representation of surface gold working electrode modification with 4-aminophenylacetic acid (CMA). WEs, CE, and RE are working electrode, counter electrode, and reference electrode, respectively.

Electrochemical deposition of 4-aminophenylacetic acid 98% (CMA) was made by using 5 mM of CMA in water with sodium nitrite 15 mM and HCl 15 mM. Both sodium nitrite and HCl solutions were added to the solution of CMA and kept in an ice bath for 15 min. This electrode preparation approach was adapted from that reported by Allongue et al. (1997) [27]. The final solution was kept at 0 °C and immediately used. The reductive absorption of the salt onto the electrode was achieved by four repetitive scans of CV between 0.3 and −1.0 V at 200 mV s^−1^. Figure 2 shows a schematic illustration of CMA deposition onto gold WEs.

### 2.5. Process for Biosensor Bio-Functionalization

A solution of EDC 0.4 M and NHS 0.1 M was made up in 1 mL of anhydrous ethanol, and the device was incubated in a 2 mL Eppendorf for 1 h at room temperature. After this time, the device was rinsed with HCl 0.1 M and dried with nitrogen stream carefully. Immediately, the CMA modified WEs were incubated in 60 µL of SA2BSA antigens at 100 µg mL^−1^ for 1 h at room temperature. Then, the microelectrodes were rinsed with PBS and incubated in 0.1% solution of ethanolamine (ETA) in PBS for 20 min at room temperature. This deactivated all remaining carboxylic activated sites. This step was necessary in order to prevent non-specific binding. At this stage, the biosensors were ready for antibodies detection.

### 2.6. Preparation of Magnetic Nanoparticles

The magnetic nanoparticles (MNPs) were prepared using the strategy that consisted of an oil-in-water magnetic emulsion as seed in the emulsion polymerization of styrene (St) and divinylbenzene (DVB). The polymerization was carried out in a 60 mL three-necked double wall glass reactor made up of glass anchor type stirrer, a reflux condenser, and a nitrogen inlet. The temperature was controlled at 70 °C by using a thermal bath. 

**Figure 3:**
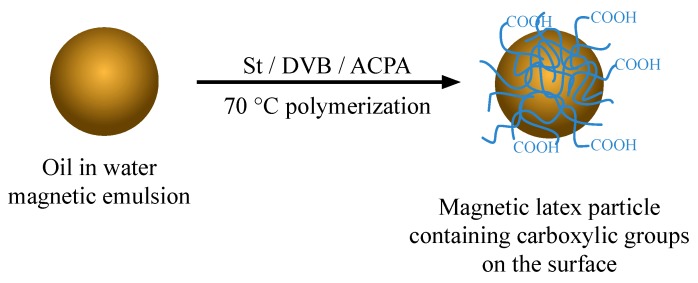
Schematic representation of magnetic nanoparticles preparation.

Firstly, the supernatant of the magnetic emulsion (50 mL) was removed after 5 min of magnetic separation. After that, the remaining magnetic particles were stabilized with the same volume of 1 g L^−1^ aqueous solution of dodecyl sodium sulfate (SDS) and introduced into the reactor. Then, the ME was purged with nitrogen for 1 h under stirring of 300 rpm to remove the residual oxygen. Afterward, a total of 1.2 mL of monomers (with ratio of 20 wt.% St and 80 wt.% DVB) was charged at once into the reactor containing the ME being kept under continuous stirring during 60 min. The radical polymerization started after adding 2 wt.% (with respect to the total weight of monomers) of 4,4′-azobis(4-cyanopentanoic acid) (ACPA) solubilized in a solution of 0.1 mol L^−1^ sodium hydroxide with SDS. The radical polymerization was conducted at constant mechanical stirring (300 rpm) and was left overnight for 24 h. A schematic representation of magnetic nanoparticles preparation is shown in Figure 3.

### 2.7. Preparation of the MNP-Ab Labels

**Figure 4:**
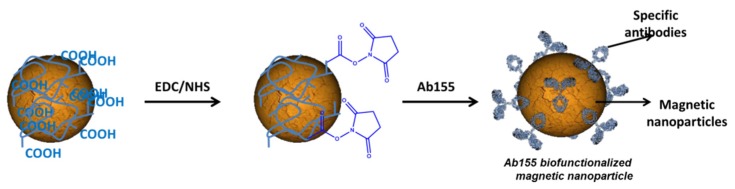
Chemical strategy for the biofunctionalization of Ab155 using carbodiimide chemistry to the magnetic nanoparticles (MNPs).

In order to increase the sensitivity of the biosensor, the antibodies Ab155 were immobilized onto MNP by the carbodiimide chemistry (Figure 4). The general procedure was based on our previous work [23]. Briefly, a vial of 1 mL of MNP was placed in a magnetic rack for 5 min to immobilize the MNP and to remove the support solution. Then, 1 mL of PBS was added in order to wash the MNP. This operation was repeated three times to ensure that all surfactants were removed. 

The carboxylic groups from the MNP were activated after treatment with a mixture of EDC 0.1 M and NHS 0.1 M and prepared in anhydrous ethanol for 90 min with continuous stirring at room temperature. After this time, the MNPs were rinsed by using a magnetic rack three times with HCl 1 mM in order to keep the activity of carboxylic acid. Then, 500 µL of a purified antibody Ab155 (100 µg mL^−1^ in PBS 10 mM) was added to the activated MNP with stirring (500 rpm at room temperature) for 2–3 hours. The Ab155 was previously purified from the antisera As155 by ammonium sulfate precipitation at 40% of total saturation. Finally, the vial of Ab155-modified MNP was washed with PBS 3 times using the magnetic rack and stored in PBS at 4 °C. The antibody reacted with the activated COOH by the free accessible lysines of the antibody, as shown in Figure 4. 

### 2.8. Magneto-ELISA for MNP-Ab155/SA7-HRP

Horseradish peroxidase (HRP) labeled with a specific enzymatic tracer SA7-HRP for Ab155 (2 µg mL^−1^ in PBS-tween) was mixed with a suspension of different concentration of the biomodified magnetic beads (100 μL, from 100 to 0 µg mL^−1^ of Ab155-MNP). The immunological reaction was allowed to proceed for 30 min at RT with vigorous shaking. The magnetic beads were then washed with PBST (150 μL, three times), and the substrate solution (100 μL, 0.01% tétraméthylbenzidine (TMB) and 0.004% H_2_O_2_ in citrate buffer 0.05M pH 5.5) was added and incubated again for 30 min at RT. The enzymatic reaction was stopped by adding 2 M H_2_SO_4_ (50 μL). Finally, the supernatants were removed from the magnetic beads and then added to a different plate for measuring the absorbance at 450 nm.

## 3. Results

### 3.1. Characterization of the Magnetic Nanoparticles

**Figure 5:**
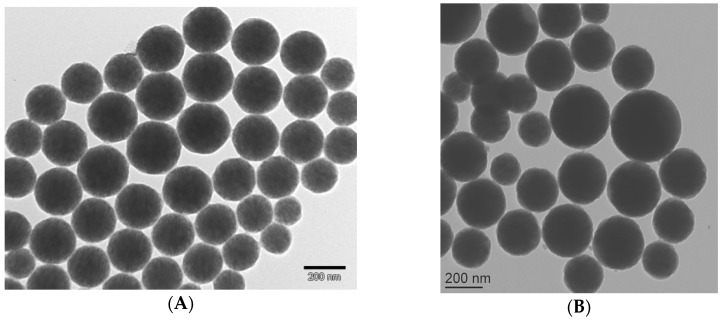
TEM images of: (**A**) magnetic emulsion, (**B**) samples (with core-shell like morphology containing COOH moieties from 4,4′-azobis(4-cyanopentanoic acid) (ACPA) obtained after seed-polymerization using 20 wt.% St and 80 wt.% divinylbenzene (DVB).

The characterization of the produced nanoparticles was performed by transmission electron microscopy (TEM), dynamic light scattering (DLS), zeta potential measurement, and thermal gravimetric analysis (TGA). Before polymerization, the average particle size of ME was approximately 237 nm [20]. As we expected, after polymerization, the average particle size increased up to 296 nm due to addition of polymer shell (See Figure 5). However, due to the presence of such a polymer shell, the values of zeta potential decreased. In a pH ranging from four to nine, the particles showed zeta potential values of −40 to −50 mV and −28 to −35 mV for magnetic emulsion and core-shell particles, respectively. The highest zeta potential values for ME was associated with the presence of the strong acid sulfate groups (−SO_4_^−^) emanating from SDS (stabilizing agent) of the magnetic emulsion. However, after polymerization, the ME surface was covered by non-charged molecules, and only ACPA could contribute to the surface charge due to the presence of COOH moieties.

**Figure 6:**
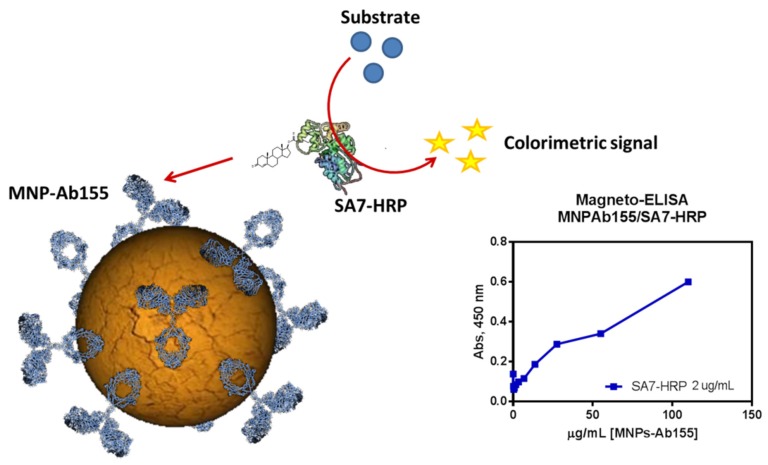
Representation of a magneto-ELISA and the results obtained for MNPs coupled with Ab155 (specific for SA7-HRP and Sulfonamides).

Once the final bioconjugates were purified, a characterization of the biofunctionality was required. The best way to know the functionality of the Ab-MNPs conjugate was by magneto-ELISA (Figure 6). This method consisted of testing the Ab-MNPs conjugate with an enzymatic tracer that had an affinity for the immobilized antibody. After a period of incubation and washing, a substrate was added in order to obtain the colorimetric signal from the enzyme, which was bound to the antibody. The antibody amount immobilized onto the surface was quantified and found to be 100 µg mL^−1^ for batch preparation. The magneto-ELISA let us conclude that not only was the immobilization was successful but also the antibody was functional and the obtained signal was specific.

### 3.2. Characterization of CMA-Modified Electrode.

**Figure 7:**
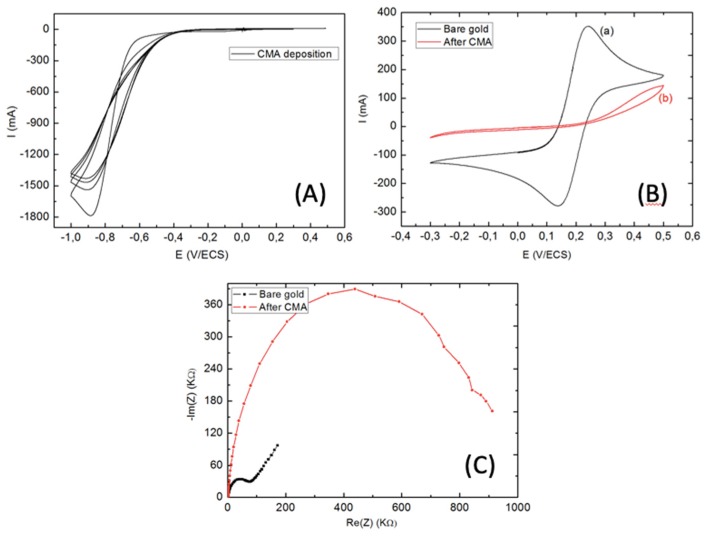
(**A**) Cyclic voltammetry (CV) for electrodeposition of CMA. Four repetitive potential cycles from 0.3 V to −1.0 V with a scan rate of 0.2 V s^−1^. (**B**) Cyclic voltammetry scan, (**C**) electrochemical impedance spectroscopy for bare gold WE (black line), and CMA immobilized onto the WE (red line).

The reductive adsorption of the salt onto the electrode was achieved by four repetitive scans of CV between 0.3 and −1.0 V at 200 mV s^−1^. The initial cycle in Figure 7A shows a broad and irreversible cathodic wave with a potential peak at −0.9 V, which indicates diazotized CMA attachment onto the gold surface by diazonium salt reduction. The cathodic current was remarkably weakened in the successive scans. These were attributed to the large passivation area of the microelectrode. This statement was confirmed by CV before and after gold WEs modification (Figure 7B), where an important decrease in peak-to-peak of the CV cycle was seen when compared to the bare gold microelectrodes.

These results were confirmed by EIS analysis before and after CMA deposition (Figure 7C). Therefore, the first semi-circle of Nyquist plot diagram corresponded to impedance of gold WEs, which was too weak when compared to CMA modified WEs. This was related to the passivation of WEs with the CMA layer, which increased the transfer charge resistance (R_tc_) between the modified WEs and the electrolyte solution.

### 3.3. Titration Assay for Ab155 to SA2BSA Coated Wes by EIS

**Figure 8:**
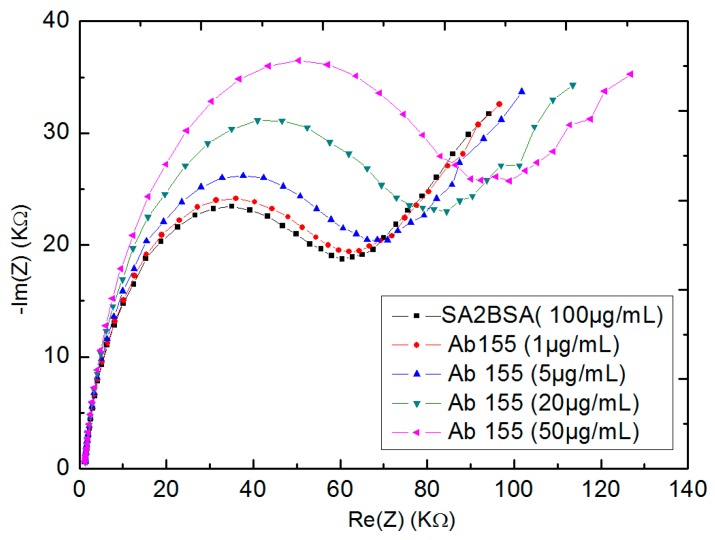
Electrochemical impedance spectroscopy (EIS) measurements of different concentrations of Ab155 with fixed concentration of SA2BSA.

Electrochemical impedance spectroscopy (EIS) was made for the coated-antigens SA2BSA gold WEs (Figure 8). Here, the first semi-circle of Nyquist plot (black line) corresponded to the coating antigens without any detection. In order to detect Ab155 antibodies, the biosensors were rinsed with PBS and incubated in 60 µL of antibodies aqueous solution of Ab155 at different concentrations ranging from 1 µg mL^−1^ to 50 µg mL^−1^ for 30 min, then rinsed with PBS and analyzed with EIS.

The subsequent Nyquist plot semi-circles were shifted from the first, indicating an increase of the transfer charge resistance (R_tc_). As the Rtc became higher, the greater the concentration of antibodies Ab155 was.

### 3.4. Competitive Assay for the Detection of Sulfapyridine (SPY) Usin SA2-BSA Coated Electrode with Ab155

After showing the interactivity of the coating antigens with different concentrations of Ab155 antibodies, the same principle of EIS detection was applied in this section by using Ab155 antibodies with another antigen sulfapyridine (SPY). The SPY is also specific to Ab155 antibodies. Here, a mixture of 20 µL of fixed concentration of Ab155 antibodies (50 µg mL^−1^) was added to 20 µL of different concentrations of SPY 40 µM, 4 µM, 2 nM, and finally 0 M (only PBS), respectively. For each concentration, the two solutions (20 µL of Ab 155 and 20 µL of SPY) were then allowed to incubate the SA2BSA modified biosensor for 30 min and kept at 4 °C. After each incubation, the surface of microelectrodes was rinsed with PBS and analyzed by EIS measurements. Here, there was a competition between SPY and SA2BSA to detect Ab155 antibodies. 

**Figure 9:**
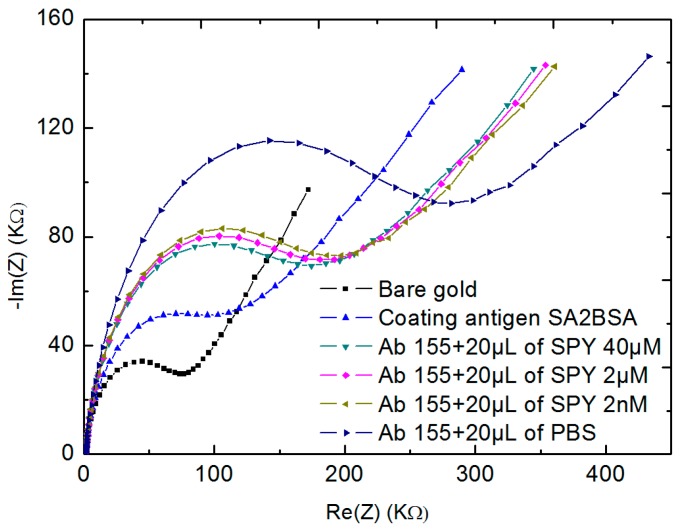
EIS measurements of Ab155 with different concentrations of SPY (40 µM, 4 µM, 2 nM) and phosphate-buffered saline (PBS) only.

Figure 9 shows the Nyquist plot semi-circles obtained for 50 µg mL^−1^ of Ab155 in the presence of different concentrations of SPY. The first and the second semi-circles corresponded to the bare gold (black line) and the coating antigens SA2BSA (blue line), respectively. After the first incubation of the biosensor within Ab155 and 40 µM of SPY (the third Nyquist plot semi-circle), we observed a shift showing a detection of Ab155 antibodies by SA2BSA. In this case, the concentration of SPY was too high, which meant a large amount of Ab155 reacted with SPY, while the rest was attached to the coating antigens SA2BSA. By decreasing the concentration of SPY, there were more antibodies Ab155 free in the solution, which have may reacted with SA2BSA. This was confirmed by the increase of Nyquist plot semi-circles. Finally, when the biosensor was incubated only in Ab155 antibodies with PBS, the totality of the antibodies reacted with the coating antigen SA2BSA, thus giving a big shift between Nyquist plot semi-circles.

### 3.5. Competitive Assay for the Detection of Sulfapyridine (SPY) Usin SA2-BSA Coated Electrode with MNP-Ab155

**Figure 10:**
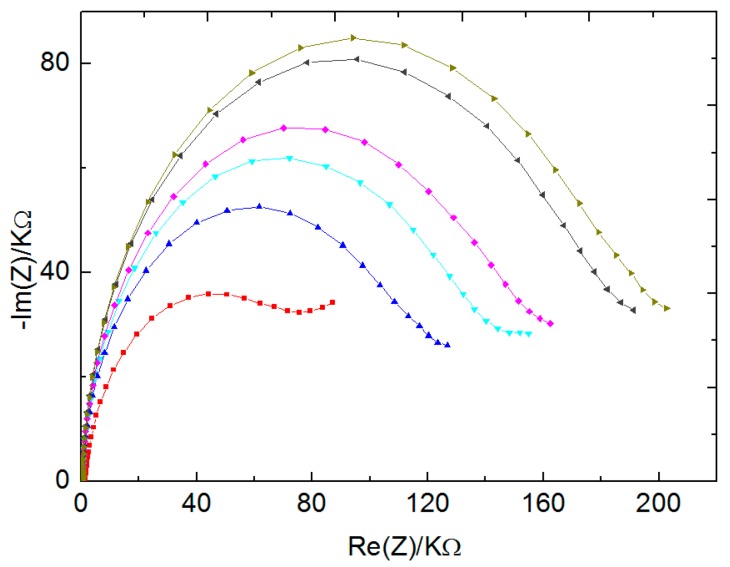
EIS measurements of fixed quantity of Ab 155 modified magnetic latex particles (20 µL) against different concentrations of sulfapyridine (SPY) (40 µM, 4 µM, 2 nM) and PBS only. Bare gold (■), SA2BSA (▲), MNP-Ab 155+40 µM SPY (▼), MNP-Ab155+ 4 µM SPY (♦); MNP-Ab 155+2 nM SPY (◀), and MNP-Ab 155+Only PBS (▶).

Firstly, the immobilization of the coating antigen with 100 µg mL^−1^ solution of SA2BSA was made onto WEs surface, as explained in Section 2.4. Afterward, the Ab155-modified MNP with fixed quantity of 20 µL was mixed with different concentrations of SPY (40 µM, 4 µM, and 2 nM) and finally only with PBS. Here, the same principle of competitive detection between SPY and SA2BSA was applied (Figure 10). By decreasing the concentration of SPY, the shift between Nyquist plot semi-circles increased. However, when compared to the competitive detection without MNP, this shift was more significant, especially for the concentrations 40 µM, 2 µM, and 2 nM. This result was associated with the high number of antibodies present on the MNP surface.

**Figure 11:**
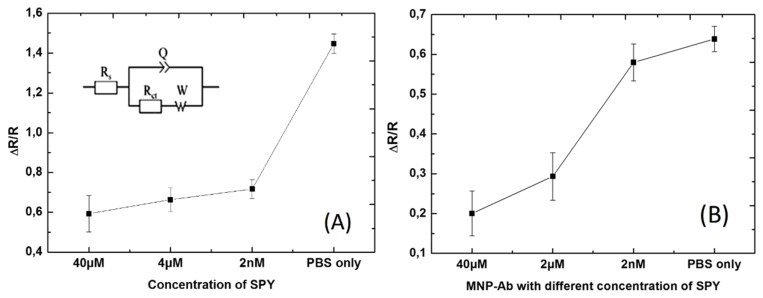
Graphical representation of fixed concentration of SA2BSA and Ab155 without magnetic nanoparticles (**A**) and with magnetic nanoparticles (**B**).

EIS measurements were repeated at least four times, as we had four Wes, and Nyquist plots were fitted for both Figure 9 and Figure 10 concerning the detection of Ab155 with and without MNP by using the equivalent circuit (Figure 11A inset). Here, Rs indicated electrolyte solution resistance, Q was the constant phase element that corresponded to the charge distribution at the interface electrode/electrolyte, R_ct_ (which was in parallel with Q) was the resistance of charge transfer, and finally, W corresponded to Warburg impedance, which is related to the diffusion phenomenon at the electrode–electrolyte interface [10,28].

After calculating fitting parameters from the applied equivalent circuit, it was clearly evidenced that there was more linearity for the biosensor with Ab155 modified MNP (Figure 11B) when compared to competitive detection without magnetic nanoparticles (Figure 11A). This result indicated that the sensitivity of the biosensor increased and provides a better understanding of the Ab155 interaction in the presence of SA2BSA and SPY at the same time. 

In this study, we had the reverse system of normal detection of biosensors. Here, as the response of the biosensors was higher, the less contamination there was, and vice versa. Indeed, when the response of the biosensor was too weak, this meant that there was an important concentration of SPY that saturated all the antibodies Ab155, such as in the case of the sample contaminated with SPY. On the other hand, if the response of the biosensor was too high, this meant that all the antibodies were collected with the coating antigens SA2BSA, such as in the presence of a sample without contamination or one that contained a weak amount of SPY.

## 4. Conclusions

In this article, we reported the sensitivity of the synthetized biosensor using the application of competitive assay by immobilized fixed concentration of SA2BSA (100 µg mL^−1^) and fixed concentration of Ab155 (50 µg mL^−1^) against different concentrations of SPY, such as 40 µM, 4 µM, 2 nM, and finally only with PBS, with and without use of magnetic nanoparticles. Using CV and EIS, the microelectrodes were characterized for the bare gold, surface-functionalized CMA, immobilized SA2BSA, and fixed concentration of MNP containing immobilized Ab155 with different concentrations of SPY.

The Nyquist plots showed a stepwise increase of R_ct_ in both cases (Ab155 with and without magnetic nanoparticles that contain immobilized Ab155 with different concentrations of SPY). However, after calculations from fitting parameters and drawing graphs, it could be clearly observed that there was more linearity while using magnetic nanoparticles compared to that without their use. This indicated the increase in sensitivity of the biosensor as compared to competitive assay without magnetic nanoparticles.
